# Fibrous MXene Aerogels with Tunable Pore Structures for High-Efficiency Desalination of Contaminated Seawater

**DOI:** 10.1007/s40820-023-01030-8

**Published:** 2023-03-21

**Authors:** Fan Wu, Siyu Qiang, Xiao-Dong Zhu, Wenling Jiao, Lifang Liu, Jianyong Yu, Yi-Tao Liu, Bin Ding

**Affiliations:** 1 Innovation Center for Textile Science and Technology, College of Textiles, Donghua University, Shanghai, 201620 People’s Republic of China; 2 State Key Laboratory Base of Eco-Chemical Engineering, College of Chemical Engineering, Qingdao University of Science & Technology, Qingdao, 266042 People’s Republic of China

**Keywords:** Fibrous MXene aerogels, Tunable pore structures, Modularized solar evaporator, Photothermal desalination

## Abstract

The seawater desalination based on solar-driven interfacial evaporation has emerged as a promising technique to alleviate the global crisis on freshwater shortage. However, achieving high desalination performance on actual, oil-contaminated seawater remains a critical challenge, because the transport channels and evaporation interfaces of the current solar evaporators are easily blocked by the oil slicks, resulting in undermined evaporation rate and conversion efficiency. Herein, we propose a facile strategy for fabricating a modularized solar evaporator based on flexible MXene aerogels with arbitrarily tunable, highly ordered cellular/lamellar pore structures for high-efficiency oil interception and desalination. The core design is the creation of 1D fibrous MXenes with sufficiently large aspect ratios, whose superior flexibility and plentiful link forms lay the basis for controllable 3D assembly into more complicated pore structures. The cellular pore structure is responsible for effective contaminants rejection due to the multi-sieving effect achieved by the omnipresent, isotropic wall apertures together with underwater superhydrophobicity, while the lamellar pore structure is favorable for rapid evaporation due to the presence of continuous, large-area evaporation channels. The modularized solar evaporator delivers the best evaporation rate (1.48 kg m^−2^ h^−1^) and conversion efficiency (92.08%) among all MXene-based desalination materials on oil-contaminated seawater.

## Highlights

- The super-elastic and robust MXene aerogels are created herein by assembling the 1D fibrous MXenes with sufficiently large aspect ratios and superior flexibility.
- The underlying regulatory mechanism and a complete diagram for the pore structure evolution of MXene aerogels are revealed for the first time, which are particularly instructive for future structure-specific designs.
- Fibrous MXene aerogels exhibit 8.3% plastic deformation at the 1000th compressions and achieve high evaporation rate (1.48 kg m^−2^ h^−1^) and light to thermal conversion efficiency (92.08%) on oil-contaminated seawater.

## Introduction

Nowadays, the increasing crises of droughts and water pollution have brought about a severe scarcity of freshwater, putting an intensifying threat to both human health and socio-economic development. Particularly, the anomalistic heatwave sweeping across the world since June 2021 has aggravated this awkward situation in areas such as Asia, Europe, and North America, ringing the alarm bell once again [1, 2]. Desalination is regarded as an effective way to relieve the pressure on freshwater supply due to the adequate seawater reserves on the earth [3]. However, the conventional desalination strategies, including multi-stage flash evaporation, reverse osmosis, and multi-effect distillation, are faced with troubles such as massive energy consumption and serious CO_2_ emissions [4]. In this sense, the photothermal desalination based on solar-driven interfacial evaporation stands out as the most attractive technique due to its potential for energy-efficient and environment-friendly applications [5–8]. Currently, carbonaceous materials and plasmonic nanoparticles are the two predominant types of photothermal materials; however, the former suffer from low light-to-heat conversion efficiency, while the latter are notorious for toxicity and instability, making both of them unsatisfactory for solar desalination [9, 10].

Two-dimensional (2D) transition metal carbides and nitrides, also known as MXenes, have emerged as a promising candidate for photothermal applications due to their excellent light-to-heat conversion efficiency (~ 100%) with a broad absorption spectrum [11]. It is worth noting, however, that the real environment for freshwater acquisition from seawater is rather complex, and oil contaminants, as a large class of inevitable impurities other than inorganic salts, would greatly reduce the desalination performance of the photothermal materials, and even make them invalid during the service time [12]. As such, when we talk about the potential of MXenes for seawater desalination, the ability to intercept the oil contaminants (liquid–liquid separation) is of the same importance as the conversion efficiency (vapor–liquid separation), which was usually overlooked in previous studies [5]. Like other 2*D* nanomaterials, unfortunately, MXenes often exist in the form of thin films composed of tightly stacked nanosheets, resulting in a densified structure as well as reduced evaporation interfaces [13]. Therefore, the MXene films are difficult to achieve, e.g., rapid liquid transport, efficient oil/water separation, and sufficient heat control, so their conversion efficiency is far below the theoretical value when dealing with actual, oily seawater [14, 15].

In this context, some pioneering works have tried to assemble 2*D* MXene nanosheets into three-dimensional (3*D*) porous aerogels, aiming to obtain more evaporation interfaces [16, 17]. However, in the present MXene aerogels, the large and relatively rigid nanosheets are randomly piled up to form irregular and disconnected macropores (25–100 μm) with compact pore walls [15, 18, 19], which fail to form ordered evaporation arrays for a boosted evaporation rate [20]. Besides, the large pore size is invalid for the interception of emulsified oil droplets since their size is much smaller (< 20 μm) [20]. As such, the evaporation interfaces of these aerogels would be easily occupied by oil slicks during the seawater evaporation process, leading to significantly undermined conversion efficiency [21, 22]. Moreover, the irregular pore structure is susceptible to distortion or even collapse since it lacks a cooperative deformation–recovery behavior to dissipate the external stress, making these aerogels unstable in long-term irradiation and tidal shock conditions [23, 24]. Therefore, how to construct MXene aerogels with highly ordered, size/shape-tunable, and mechanically robust pore structures is at the forefront of research for the integrated purification and desalination of oily seawater, which are expected to not only provide abundant evaporation interfaces, but also realize effective oil sieving and stable service [25].

**Scheme 1:**
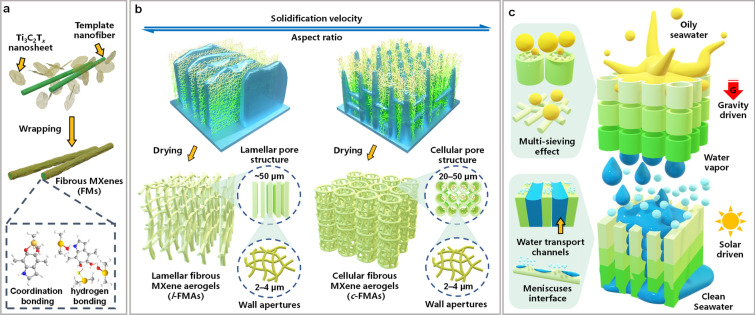
Schematic illustration of a modularized solar evaporator based on fibrous MXene aerogels with tunable pore structures for high-efficiency desalination of oily seawater

Compared to 2*D* nanosheets, one-dimensional (1*D*) fibrous nanomaterials possess large aspect ratios, superior flexibility, and plentiful link forms (bonding, lapping, and winding), all of which are favorable for controllable assembly into more complicated pore structures [26, 27]. Moreover, one of the most attractive is the development of this fibrous nanomaterials showing activable properties on-demand [28]. Therefore, it is a fascinating concept, and also a daunting challenge, to construct 3*D* aerogels with well-organized pore structures based on 1*D* fibrous MXenes (FMs) as the building blocks. Recently, 2*D* MXene nanosheets were transformed to nanoscrolls or nanotubes by cutting or curling [29]. However, the length of these nanoscrolls or nanotubes was limited by the size of the MXene nanosheets (< 10 μm), whose small aspect ratios and poor continuity limited their potential for delicate 3*D* assembly [30]. To tackle this challenge, we envision creating 1*D* FMs with sufficiently large aspect ratios by a nanofiber-templating strategy (Scheme 1a and Scheme S1a). Through ice-crystal-assisted assembly, fibrous MXene aerogels (FMAs) are constructed with arbitrarily tunable cellular/lamellar pore structures, laying the basis for structure-specific applications (Scheme 1b and Scheme S1b).

Furthermore, an interesting concept of modularized solar evaporator, based on the FMAs with different pore structures, is conceived (Scheme 1c). In this modularized solar evaporator, the oily seawater first passes through the cellular FMAs (*c*-FMAs) by gravity, where the emulsified oil droplets are promptly and efficiently intercepted due to the multi-sieving effect achieved by the omnipresent, isotropic wall apertures (2–4 μm) together with underwater superhydrophobicity, showing an excellent oil/water separation effect (TOC content < 10 mg L^−1^). The purified seawater is then transported to the surface of the lamellar FMAs (*l*-FMAs) by capillary action, and is changed into water vapor under solar irradiation. The *l*-FMAs exhibit superior light absorbance (> 93.5%) in the full spectrum, as well as strikingly high evaporation rate (1.48 kg m^−2^ h^−1^) and conversion efficiency (92.08%) under one-sun irradiation due to the presence of continuous, large-area evaporation channels enabling a strong water pumping ability and multi-meniscuses air/water evaporation. Moreover, both FMAs have stable porous frameworks, and can withstand 1000 large-scale compression cycles without obvious deformation. Finally, the capacity of this modularized solar evaporator in reducing the CO_2_ emissions is simulated based on a model accounting for the statistical data of the whole China, demonstrating its huge potential for energy-saving freshwater acquisition.

## Experimental Section

### Creation of 1D FMs with Different Aspect Ratios

Potassium persulfate was dissolved in a dibasic sodium phosphate/citric acid buffer (pH = 7) at a molar ratio of 1:1, and dopamine hydrochloride was dissolved in this buffer. The SiO_2_ TNFs, prepared by a combination of sol–gel method and electrospinning as reported by us previously [31], were immersed in this buffer at a mass ratio of 1:1 for 1 h to obtain an active surface (Scheme S1a). The TNFs were dispersed by a shearing machine to control the aspect ratios, and the shearing parameters were presented in Table S1. After that, a colloidal solution (2 mg mL^−1^) containing Ti_3_C_2_T*x* nanosheets, and the dispersion of the TNFs (1 mg mL^−1^) were mixed, and kept under stirring for 30 min. Then, the mixture was centrifuged at 5000 rpm for 8 min, the solid residue was washed with deionized water, and centrifuged at 5000 rpm for another 8 min. The reaction was repeated 1, 5, and 10 times to obtain FMs with different surface structures.

### Construction of FMAs with Tunable Pore Structures

In a typical experiment for preparing FMAs with a bulk density of 8 mg cm^−3^, 1 g of FMs were dispersed in 125 g of deionized water. Then, 1.04 g of SiO_2_ sol was added, and homogeneously dispersed by using a high-pressure homogenizer. Finally, the homogenized dispersion was frozen, and transferred to a lyophilizer for vacuum freeze-drying for 48 h to obtain FMAs. Additionally, the aspect ratios of the FMs and the solidification velocity were varied to investigate their influences on the pore structures (Scheme S1b).

### Ice Nucleation Experiment

The formation temperatures of ice crystals in the dispersions containing the FMs with different aspect ratios were measured by DSC. All samples were set to cool from 10 to − 80 °C at a cooling rate of 10 °C min^−1^, held for 5 min, and then rewarmed to 10 °C at a heating rate of 15 °C min^−1^.

### Design and Fabrication of Modularized Solar Evaporator

A modularized solar evaporator was designed which consisted of 3 parts: an oily seawater purification chamber, a solar desalination chamber, and a freshwater storage chamber. The core of the purification chamber was a tubular separator prepared by polymethyl methacrylate (PMMA), where the *c*-FMAs were fixed in the middle of the tubular separator. The oily seawater was separated by the *c*-FMAs by gravity. Then, the purified seawater entered the solar desalination chamber, where the *l*-FMAs were fixed and connected to the purified seawater by filter paper sheets. The purified seawater was continuously transported to the surface of the *l*-FMAs by capillary action. Under solar irradiation, the *l*-FMAs changed seawater into water vapor, which was condensed on the surface of a polyethylene terephthalate (PET) plate and flowed into the freshwater storage chamber.

### Oil/Water Separation Experiment

Different oil-in-water emulsions were prepared by mixing oils, e.g., trichloromethane, petroleum ether, *n*-hexane, and hexadecane, with simulated seawater at an oil/seawater volume ratio of 1:9. Then, the mixtures were sonicated for 30 min under 600 W to obtain the oil-in-water emulsions. The oil/water separation through the modularized solar evaporator was performed on a dead-end filtration device. The *c*-FMAs were sandwiched between a glass vessel and a *φ*20 mm funnel base. The newly prepared oil-in-water emulsions were poured into the separation cell, and separated under gravity. The flux was determined by calculating the filtrate volume in 1 min. The gravitational pressure could be adjusted by controlling the height of the emulsion column. The separation products were analyzed by a TOC analyzer.

### Photothermal Desalination Experiment

The photothermal desalination experiment was conducted by using a solar simulator with a simulated solar flux of 1 kW m^−2^. The *l*-FMAs (1 × 1 cm^2^) were fixed on the top of the PMMA container, and hydrophilic nonwovens connected the channels in the *l*-FMAs with seawater to continuously feed the seawater via capillary force. The experiment was conducted at a temperature of 25–26 °C and a humidity of 55–60%. The size of light spot was controlled to be equal to that of the *l*-FMAs, ensuring the accuracy of the experiment. The solar flux was measured by a solar power meter. The mass change during desalination was recorded by an electronic balance, and the temperature was measured by an infrared thermal imager.

The solar steam efficiency *η* was calculated by the following formula:

1 $$\eta = mh_{v} /Aq_{solar }$$

where ${\text{m}}$ was the evaporation rate, ${\text{h}}_{v}$ was the total enthalpy of water, which was composed of phase change enthalpy and sensible heat, $A$ was the surface area of the absorber facing the sun, and ${\text{q}}_{solar}$ was the solar flux per area.

## Results and Discussion

### Creation and Characterization of 1D FMs

**Fig. 1:**
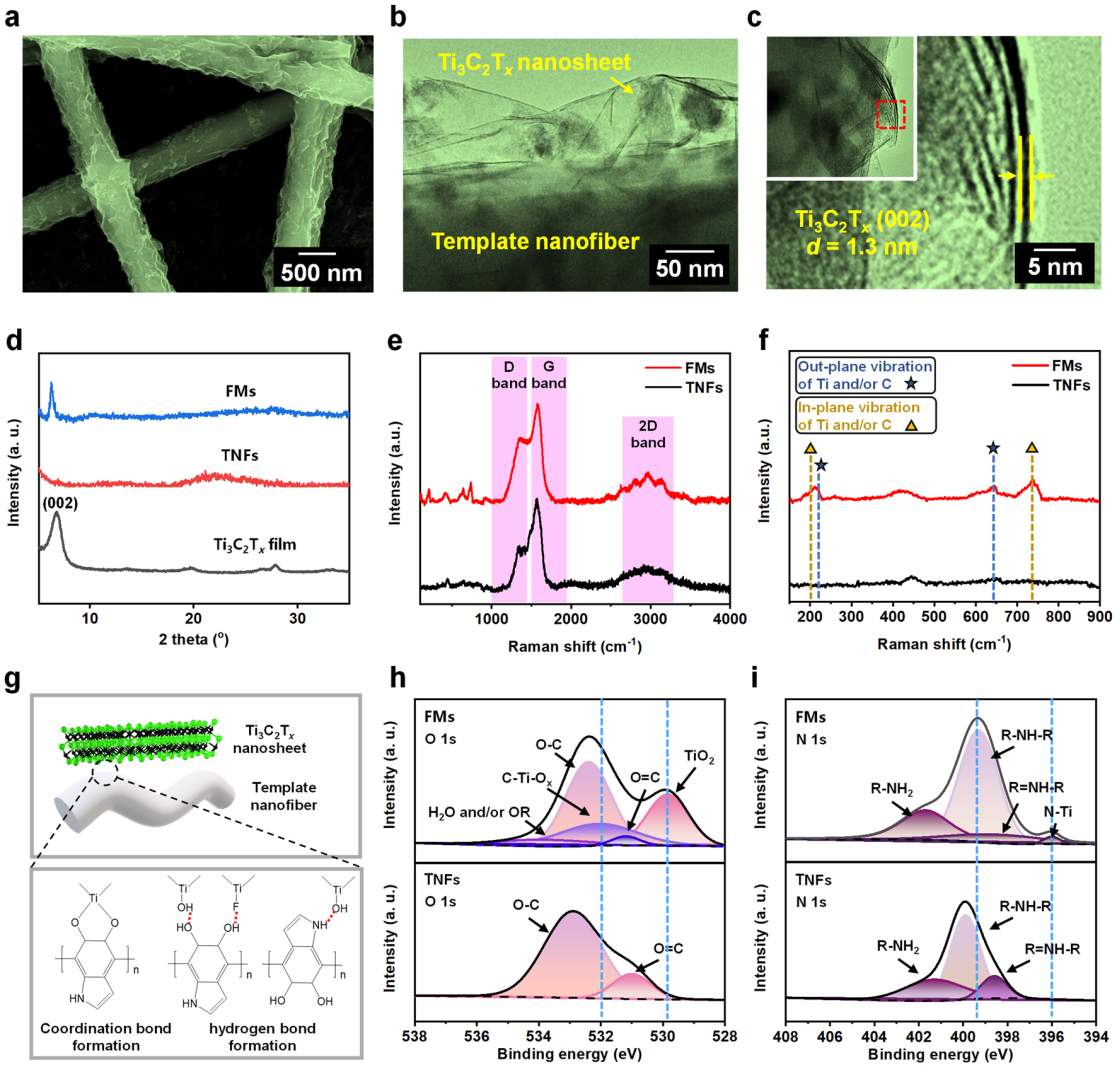
Characterization of 1D FMs. (**a**) SEM image of 1D FMs. (**b**, **c**) TEM images of an individual TNF wrapped by few-layer Ti_3_C_2_T_*x*_ nanosheets on the surface. (**d**) XRD patterns of FMs, TNFs, and a Ti_3_C_2_T_*x*_ film. (**e**, **f**) Raman spectra of FMs and TNFs. (**g**) Proposed interactions between TNFs and Ti_3_C_2_T_*x*_ nanosheets through coordination/hydrogen bonding. (**h**, **i**) O 1*s* and N 1*s* XPS spectra of FMs and TNFs

To create 1D FMs with sufficiently large aspect ratios, we propose a facile strategy by wrapping ultrathin Ti_3_C_2_T_*x*_ MXene nanosheets onto template nanofibers (TNFs) through surface-chemistry-mediated interactions. At first, flexible TNFs were prepared by electrospinning according to our previous report [31]. Then, an active surface rich in –OH and –NH_2_ groups was formed on the TNFs by the polymerization reaction of dopamine (Fig. S1a), which laid the basis for the spontaneous attraction of the Ti_3_C_2_T_*x*_ nanosheets (Fig. S1b) through coordination/hydrogen bonding to form 1D FMs (Fig. 1a) [32]. It is noteworthy that there should be a repulsive force between the Ti_3_C_2_T_x_ and polydopamine, even if the presence of secondary amine and tertiary amine in polydopamine might weaken this repulsion [33, 34]. The energy-dispersive *X*-ray spectroscopy (EDS) demonstrates uniform and complete wrapping of an individual nanofiber by the Ti_3_C_2_T_*x*_ nanosheets from the perspective of elemental composition (Fig. S1c). The detailed characterization of the FMs was realized by transmission electron microscopy (TEM), as shown in Fig. 1b, c, which discloses few-layer nanosheets with a *d*-spacing of 1.3 nm corresponding to the (002) crystal plane of Ti_3_C_2_T_*x*_ [35]. Besides, the *X*-ray diffraction (XRD) pattern of the FMs also reveals the (002) characteristic peak of Ti_3_C_2_T_*x*_ (Fig. 1d).

Figure 1e compares the Raman spectra of the FMs and TNFs. Different from the TNFs, the FMs have a mountainous region in the range of 100–800 cm^−1^ corresponding to the in-plane and out-plane vibrations of Ti and *C* atoms in Ti_3_C_2_T_*x*_, respectively [36]. The two peaks appearing at around 200 cm^−1^ represent the out-plane and in-plane vibrations of Ti and *C* atoms, respectively [36]. Besides, the peaks at around 650 and 740 cm^−1^ are assigned to the out-plane and in-plane vibrations of C atom (Fig. 1f), which demonstrates that the wrapping process did not affect the surface chemical environment of the Ti_3_C_2_T_*x*_ nanosheets [36]. Meanwhile, the sharp peaks in the 2*D* band of the FMs are also characteristic of few-layer Ti_3_C_2_T_*x*_ nanosheets [37]. Next, we would like to elucidate the interactions between the Ti_3_C_2_T_*x*_ nanosheets and the TNFs [37]. It is known that the –OH and –NH_2_ groups in dopamine may bind to multivalent metal ions via coordination bonding [38], and the –O and –F terminals of Ti_3_C_2_T_*x*_ may also bind to the –OH and –NH_2_ groups via hydrogen bonding. Both interactions would become the driving force for the wrapping of the Ti_3_C_2_T_*x*_ nanosheets onto the TNFs (Fig. 1g) [39]. To confirm the formation of the coordination bond, the O 1*s X*-ray photoelectron spectra (XPS) are presented in Fig. 1h, and the existence of a C–Ti–O_*x*_ bond at 531.0 eV arising from the catechol–Ti coordination bond is observed on the FMs [40]. Besides, the binding energy of the primary amine peak in the FMs is decreased by 0.5 eV compared to that of the TNFs, indicating the presence of hydrogen bonding between the active surface and the terminals (–O and –F) of the Ti_3_C_2_T_*x*_ nanosheets (Fig. 1i) [41]. Moreover, a new N–Ti peak appears at ~ 396.0 eV, resulting from the bonding of amine to the Ti sites [42].

A further investigation was conducted to clarify the influences of some key parameters on the wrapping effect. At first, the Ti_3_C_2_T_*x*_ nanosheets with lateral sizes of ~ 2 μm, ~ 500 nm, and ~ 200 nm were obtained by fractional centrifugation at different speeds, and reacted with the surface-activated TNFs separately. As shown in Fig. S1d, the nanosheets of ~ 2 μm were hard to bend at a large curvature due to high Young's modulus [43], so the TNFs remained naked to a large extent without full coverage. On the other hand, the small nanosheets (~ 200 nm) were more prone to aggregation, resulting in complete yet non-uniform coverage along the TNFs. Therefore, only the nanosheets with a medium size (~ 500 nm) were suitable for forming an even wrapping layer on the whole surface of the TNFs. Moreover, we evaluated the influence of the reaction times by using the nanosheets of ~ 500 nm, and the results demonstrate that the coverage thickness positively correlated to the reaction times. Therefore, the subsequent experiments were carried out based on the FMs with the optimum structure (reaction times = 5).

### Construction and Characterization of FMAs with Tunable Pore Structures

**Fig. 2:**
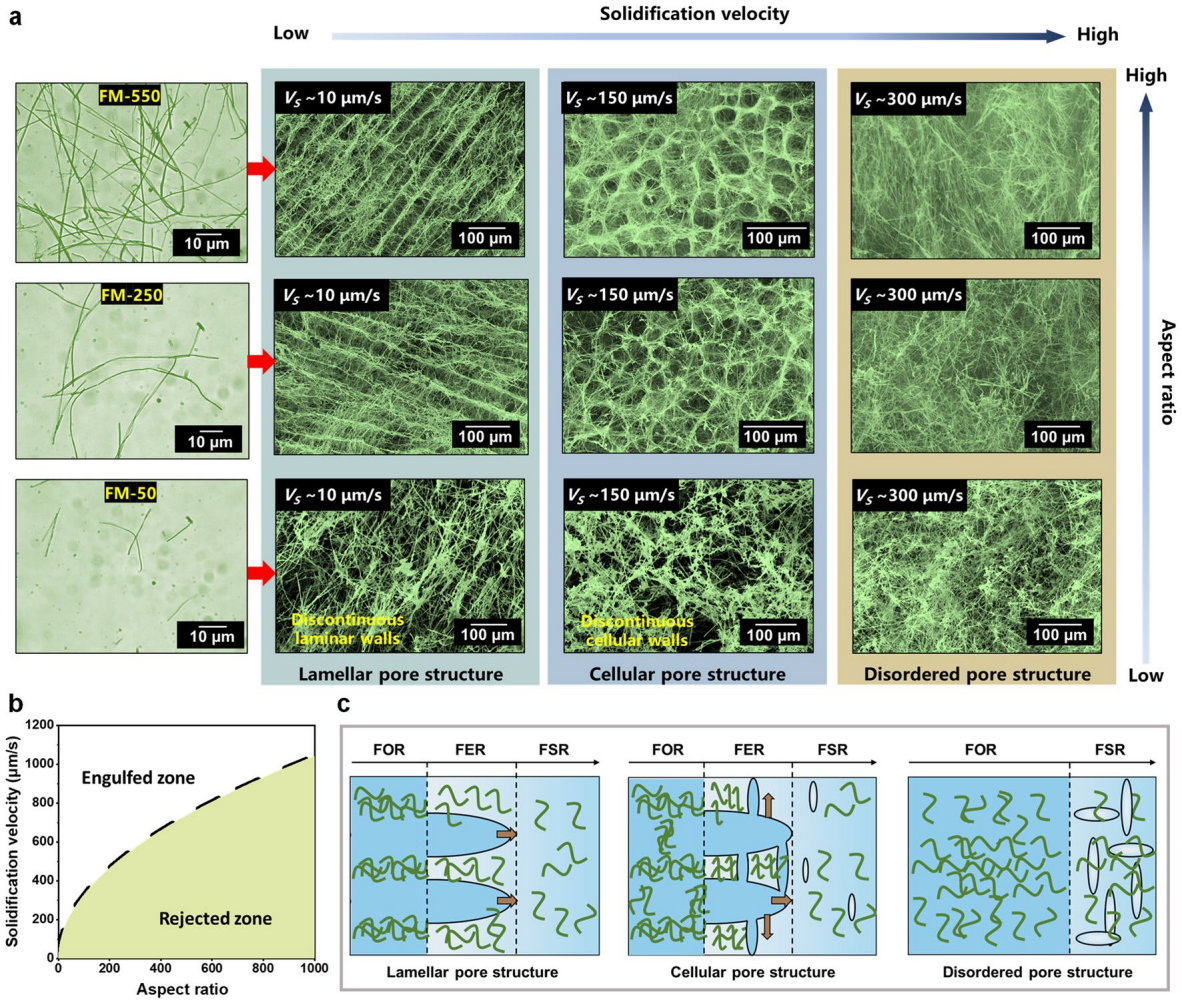
Construction and structure evolution of FMAs. (**a**) FMAs with tunable pore structures by regulation of aspect ratio and solidification velocity. (**b**) Engulfed zone and rejected zone for FMs (Dotted line, calculated critical velocity ${v}_{c}$ from Eq. 4. (**c**) Schematic illustration of ice crystal growth in different regions

It is of the utmost importance to build up the regulation principles for the controlled assembly of nanofibers into aerogels with a target architecture, which was never reported before. We began this exploration by making clear the influences of two key parameters, i.e., solidification velocity and aspect ratio. At first, the FMs were subjected to shear dispersion and centrifugal classification (Table S1), and were named FM-50, FM-250, FM-550, and FM-950 according to different aspect ratios. The classified FMs were then freeze-dried at different solidification velocities to form FMAs, whose microstructures are displayed in Fig. 2a. It is found that when the aspect ratio was too large (FM-950), the resulting FMAs exhibited a disordered pore structure regardless of the solidification velocity (Fig. S2a). In other cases, increasing the solidification velocity transformed the FMAs from lamellar to cellular and eventually to disordered pore structure. The only differences lay in the fact that the pore structure of the FMAs constructed from FM-50, being it lamellar or cellular, was discontinuous and imperfect. In contrast, both FM-250 and FM-550 maintained complete lamellar and cellular pore structures, due to their excellent continuity arising from sufficiently large aspect ratios. The above results indicate that there indeed exist a regularity with which the pore structure is determined by the feature parameters of the FMs and ice crystals.

To understand the mechanism for the distinct assembly behaviors of different FMs, we tried to work out a structure-transition diagram. We first conducted the force analysis on an individual FM in the dispersion by simplifying it to a cylinder (Fig. S2b). From the thermodynamics perspective, the balance of the two forces acting on the FM determined whether it was repelled or trapped by the freezing front interface [44]. The repulsive force, called ${F}_{R}$, came from the molecular van der Waals interactions at the fiber–water interface [45]:

2 $$F_{R} = 2l\Delta \gamma_{0} \left( {\frac{{a_{0} }}{d}} \right)^{n}$$

where $l$ was the length of the FM, *Δγ*_0_ was the dispersion system's free energy, *a*_*0*_ was the average distance between molecules in the liquid layer, and $d$ was the thickness of the liquid layer between the FM and the solid–liquid interface.

Besides this repulsive force, the FM also experienced an attractive force (${F}_{A}$) due to the viscous drag, which pushed the FM towards the solid–liquid interface:

3 $$F_{A} = \frac{{\rho v^{2} r^{2} \left( {1 + \frac{10}{{R_{e}^{0.75} }}} \right)}}{ 2d}$$

where $\rho$ was the density of water, $v$ was the solidification velocity, and $r$ was the diameter of the FM. The formula ${F}_{A}$ was derived from the Oseen solution for the drag of a cylinder in the crossflow [46]. When ${F}_{R}$ and ${F}_{A}$ were equilibrated, a critical solidification velocity (${v}_{c}$) of the freezing front could be calculated where the FM rejection or entrapment took place [47]:

4 $$v_{c} = \left( {\frac{{4l\Delta \gamma_{0} d}}{{\rho r^{2} \left( {1 + \frac{10}{{R_{e}^{0.75} }}} \right)}}} \right)^{\frac{1}{2}}$$

Therefore, a force map was plotted based on the critical solidification velocity (Fig. 2b). When the solidification velocity was lower than ${v}_{c}$, the freezing front would reject the FMs and kept them in the liquid phase. The accumulation of the FMs between the ice crystals provided the possibility of forming a lamellar pore structure. When the solidification velocity was higher, the freezing front would engulf a certain fraction of FMs and keep them in the ice crystals, which created bridges between rejected fibrous walls, forming the interleaving pore structure. (Fig. S2c).

On this foundation, we divided the assembly processes into three regions, *i.e.*, frozen region (FOR), freezing region (FER), and fiber suspension region (FSR) to explain the formation of the lamellar, cellular, and disordered pore structures (Fig. 2c). The FMs could act as the heterogeneous nucleation sites when the dispersion system reached an appropriate supercooling degree. The ice crystals usually grew perpendicularly to the cold source at a slow solidification velocity, which could not form enough supercooling degrees in FER and FSR to meet the prerequisites for heterogeneous nucleation, forming the lamellar pore structure. When the supercooled state occurred in FER or FSR by increasing the solidification velocity, new ice crystals could nucleate uniformly around the original columnar ice crystals. The strong anisotropy of the ice crystal growth kinetics led to rapid growth of favorable ice crystals [48], so that many “ice bridges” were formed between the original, vertically growing ice crystals, forming the cellular pore structure. The differential scanning calorimetry (DSC) results show that decreasing the aspect ratio of the FMs could increase the freezing temperature (Fig. S2d, e), which supported the phenomenon that the FMs with small aspect ratios were easier to form the cellular pore structure in Fig. 2b. When the solidification velocity was extremely high, the boundaries of the three regions became blurred. The supercooled state covered the whole dispersion system immediately, along with the occurrence of the crystallization sites around the FMs, which fixed the dispersed FMs and retained their original state accompanied by the rapid growth of ice crystals, forming the disordered pore structure.

**Fig. 3:**
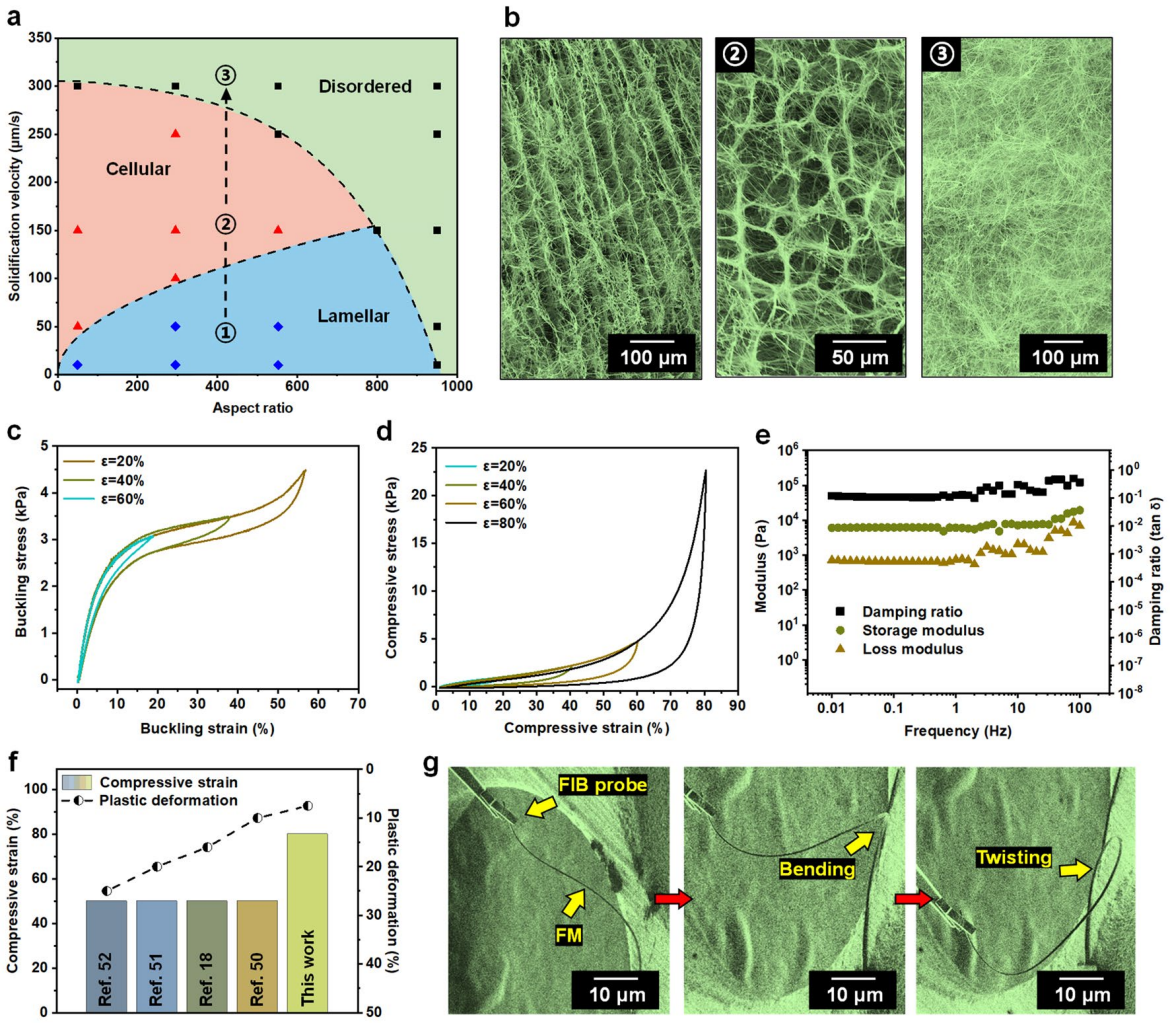
Structure transition diagram and mechanical properties of FMAs. (**a**) Structure-transition diagram of FMAs. (**b**) SEM images of FMAs corresponding to points 1, 2, and 3 in **a**. (**c**) Buckling stress–strain curves of *c*-FMAs during buckling–recovery cycles with increasing strain (0 to 60%). (**d**) Compressive stress–strain curves of *c*-FMAs during compressive–recovery cycles with increasing strain (0 to 80%). (**e**) Storage modulus, loss modulus, and damping ratio of *c*-FMAs *vs.* frequency (0.1–100 Hz). (**f**) Comparison of compressive strain and plastic deformation of *c*-FMAs and the present MXene aerogels. (**g**) In situ bending of an individual FM by using an FIB-SEM system

We can now plot a semi-empirical diagram (including lamellar, cellular, and disordered zones) for the pore structure transition of the FMAs based on the experimental results (Fig. 3a). The repulsive or phagocytosis behavior of the ice crystals on the FMs determined the mutual transition of the lamellar and cellular pore structures. The heterogeneous nucleation and fast freezing interactions also made it possible for the disordered pore structure to emerge. To validate this diagram, we further designed a dynamic pathway (1–2–3) crossing the three zones, and examined its pore structure transition process (Fig. 3b). As expected, lamellar, cellular, and disordered pore structures occurred in sequence by increasing the solidification velocity, consistent with the zones corresponding to points 1, 2, and 3. This experimental feedback demonstrates that our structure-transition diagram is indeed instructive for designing the fibrous aerogels for structure-specific applications.

The mechanical properties of the pore structures are particularly vital to the stability as well as service life of the aerogels [49]. Therefore, we further tested the strength and modulus of the *c*-FMAs and *l*-FMAs, as well as their relationship with different pore structures. As illustrated in Figs. 3c and S3a, the bending process could be divided into three stages: (I) elastic-like deformation zone (0 < ε ≤ 5%), (II) plateau zone (5% < ε ≤ 50%), and (III) densification zone (ε > 50%). In the first two stages, the *c*-FMAs and *l*-FMAs exhibited similar mechanical behaviors, yet the former entered the densification zone much earlier and had a significant increase in bending stress due to the adequate supporting sites parallel to the stress direction. Compared to the present MXene aerogels, the unique fibrous frameworks endowed our FMAs with good bending resilience, which could withstand a large bending deformation (60%) without fracture or plastic deformation. Moreover, in contrast to the present MXene aerogels, our FMAs manifested desirable compressive performance, which could withstand a considerable strain (80%) without failure (Figs. 3d and S3b) [49–51]. Interestingly, the *l*-FMAs exhibited lower compressive stress and greater tendency to drop in pressure at the beginning of the unloading process than the *c*-FMAs, probably due to the lack of effective interlayer support, which demonstrated that the former was softer, while the latter were like an elastomer. Moreover, the plastic deformation of the *c*-FMAs and *l*-FMAs was less than 10% after 1000 compression cycles under a strain of 60% (Fig. S3c, d), indicating that they possessed good impact resistance. The superelasticity, in combination with the shallow plastic deformation, made our FMAs reliable under various mechanical shock conditions, outperforming the present MXene aerogels (Fig. 3f) [18, 50–52]. Additionally, the storage modulus, loss modulus, and damping ratio of our FMAs remained stable as the compressive frequency increased from 0.1 to 10 Hz (Fig. 3e). Finally, our FMAs also exhibited excellent fatigue resistance at low (− 100 °C) and high (400 °C) temperatures, as shown in Fig. S3e, highlighting their potential to serve in complex temperature environments.

Indeed, the mechanical behaviors of a macroscopic material depend on its building blocks [53]. Thus, the superior resilience of our FMAs is related to individual FMs and the fibrous pore structure. As shown in Fig. 3g, the FMs could respond to the external stress in different directions and achieve effective stress dissipation through bending and twisting to maintain the stability. Therefore, the external stress could be transferred along the cellular framework during the stressing process when the *c*-FMAs were taken as an example. Accordingly, the pore walls composed of the FMs were inclined to bend to dissipate the external stress and gain a potential energy, in which the deformation of individual FMs and the sliding between them became critical. When the external stress was relieved, the energy stored in the FMs and pore walls drove the *c*-FMAs back to their original state (Fig. S3f).

### Oil/water Separation and Photothermal Desalination Performance of Modularized Solar Evaporator

**Fig. 4:**
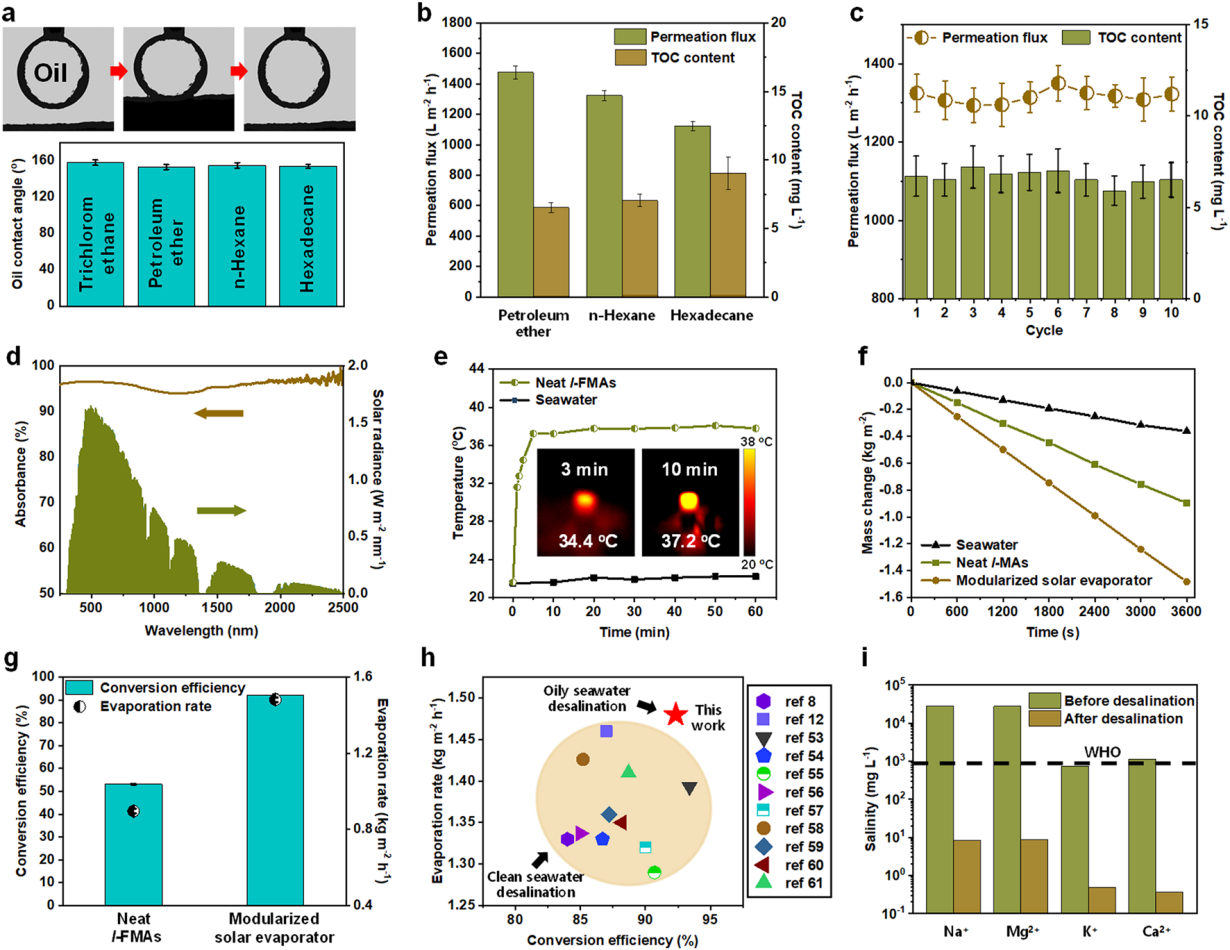
Oil/water separation and photothermal desalination performance. (**a**) Surface wettability of *c*-FMAs. (**b**) Permeation fluxes and TOC contents of *c*-FMAs for treating different oil-in-water emulsions. (**c**) Change of permeation flux and TOC content of *c*-FMAs for treating petroleum ether over 10 cycles. (**d**) Solar spectrum (AM 1.5) and UV–vis absorbance of *l*-FMAs in the range of 200–2500 nm (wet state). (**e**) Surface temperature variation profiles of *l*-FMAs under one-sun irradiation. (**f**) Comparison of mass changes of water over time of neat *l*-FMAs and modularized solar evaporator under one-sun irradiation. (**g**) Comparison of evaporation rates and conversion efficiencies of neat *l*-FMAs and modularized solar evaporator. (**h**) Comparison of conversion efficiencies of modularized solar evaporator and previously reported MXene-based desalination materials under one-sun irradiation. (**i**) Concentrations of Na^+^, Mg^2+^, K^+^, and Ca^2+^ in a simulated seawater sample before and after desalination

By integrating the *c*-FMAs and *l*-FMAs, we designed a modularized solar evaporator to overcome the difficulty in the photothermal desalination of oily seawater (Fig. S4). In this modularized solar evaporator, the oily seawater first passed through the *c*-FMAs by gravity, whose surface wettability was evaluated by the underwater oil contact angle (UWOCA) characterization, as shown in Fig. 4a. The pre-loaded oil droplet slowly approached the *c*-FMAs, followed by extrusion deformation at the contact surface. Interestingly, when the oil droplet left the *c*-FMAs’ surface, it quickly returned to the spherical shape, indicating a good anti-adhesive property of the *c*-FMAs. This special wettability was mainly attributed to the difference in the adhesion work of water and oil to the *c*-FMAs, which made it difficult for the oil droplets to enter the pore channels occupied by water [24]. Therefore, the *c*-FMAs exhibited underwater superhydrophobicity with all UWOCAs above 153° for various types of oils with different surface tensions and viscosities. Then, we selected different oil-in-water emulsions for evaluating the separation performance of the *c*-FMAs under low gravity (~ 1 kPa), as shown in Fig. S5a. The *c*-FMAs exhibited high water fluxes above 1100 L m^−2^ h^−1^ at an extremely low drive pressure, and the total organic carbon (TOC) contents in the purified seawater were < 10 mg L^−1^ (Fig. 4b), which benefited from the multi-sieving effect (Figs. S5b and S6a). Indeed, the differences in TOC contents and water fluxes of the oil-in-water emulsions were caused by different viscosities and oil concentrations [54]. Additionally, we performed the circulatory separation tests on the *c*-FMAs. As displayed in Fig. 4c, the water flux and separation efficiency were maintained at a high level after several separation cycles, reflecting excellent stability and serviceability of the *c*-FMAs.

The purified seawater was then desalinated by the *l*-FMAs, which worked as the solar panels and provided multi-meniscuses interfaces, enhancing the evaporation performance significantly (Fig. 6b). The lamellar pore walls triggered substantial light scattering and multiple internal reflections of the incident light [55, 56], thus trapping the incident light within the *l*-FMAs and ensuring superior light absorbance above 93.5% in the entire solar spectrum (Fig. 4d). Moreover, the *l*-FMAs could achieve a rapid self-warming effect with the surface temperature rising to 37.1 °C within 4 min (Fig. 4e), and possessed a low thermal conductivity in both dry (0.0372 W m^−1^ K^−1^) and wet (0.4822 W m^−1^ K^−1^) states (Fig. 4c, d), which suppressed the heat losses and laid the foundation for effective solar vapor production [57–59]. We further measured the solar-driven evaporation curve of the modularized solar evaporator to calculate the evaporation rate, and neat *l*-FMAs were selected as the control (Fig. 4f). Although neat *l*-FMAs had excellent photothermal conversion performance theoretically, the actual evaporation rate (0.8963 kg m^−1^ h^−1^) and light-to-heat conversion efficiency (53.13%) were significantly hampered in the desalination of oily seawater. In contrast, the modularized solar evaporator could overcome this limitation, and achieve an average evaporation rate as high as 1.482 kg m^−1^ h^−1^ with a light-to-heat conversion efficiency up to 92.08% under one-sun irradiation (Fig. 4g). Note that these values are even better than those of the previously reported MXene-based desalination materials dealing with clean seawater (Fig. 4h) [11, 15, 60–68]. Besides, the durability of the modularized solar evaporator is critical to its service life in harsh environments with strong irradiation and high salinity, which was evaluated by a cyclic evaporation test. As shown in Fig. S5e, the modularized solar evaporator maintained a high level of water evaporation (1.462 kg m^−1^ h^−1^) after 10 cycles (1 h for each cycle). Finally, the inductively coupled plasma spectrometry was used to monitor the seawater before and after desalination. The concentrations of all target metal ions decreased by 3 to 4 orders of magnitude after desalination, and all indicators were significantly lower than the safe salinity levels defined by the World Health Organization (Fig. 4i).

To quantitatively evaluate the potential impact of the modularized solar evaporator on the CO_2_ reduction, China, a large agricultural and industrial country with abundant water consumption, was chosen as the simulation target. We assumed an extreme scenario in which the freshwater was obtained exclusively from seawater, and collected the empirical data on the power generation structure [69], the industrial–agricultural water consumption (China Water Resources Bulletin 2021), and the electricity consumption of desalination technology to calculate the year-round CO_2_ outcome. Moreover, the reverse osmosis technology, as the main desalination technology in China currently (accounting for 82.56% of the overall desalination projects) [70], was involved in this calculation as the major comparison (more details regarding the calculation data can be found in Note S1 and Table S2). We calculated the annually averaged CO_2_ reduction over different regions by using the modularized solar evaporator, which could reduce 204.26 Mt and 664.23 Mt of CO_2_ emissions in industry and agriculture, respectively (Fig. S7). Moreover, it was expected that the modularized solar evaporator could be deployed in the Pacific Rim, Atlantic Rim, and Mediterranean Sea to separate and desalinate oily seawater by gravity and solar energy, and supply distilled water directly to the surrounding areas, thus alleviating the shortage of freshwater and contributing to the CO_2_ reduction target.

## Conclusions

In summary, 1*D* FMs with sufficiently large aspect ratios were created from 2*D* MXene nanosheets by a nanofiber-templating strategy. Through ice-crystal-assisted assembly, 3*D* FMAs with tunable pore structures were constructed, and a structure-transition diagram was plotted which was particularly instructive for designing the structure-specific fibrous aerogels. Additionally, a modularized solar evaporator based on the FMAs with different pore structures were designed, which exhibited superior performance in the desalination of contaminated seawater. The evaporation rate and conversion efficiency of this modularized solar evaporator were even better than those of the previously reported MXene-based desalination materials dealing with clean seawater. Moreover, the well-organized pore structures of our FMAs endowed them with excellent mechanical properties, ensuring stable service in long-term irradiation and tidal shock conditions. Finally, the capacity of this modularized solar evaporator in reducing the CO_2_ emissions was simulated based on a model accounting for the statistical data of the whole China, demonstrating its huge potential for green freshwater acquisition.

### Supplementary Information

Supplementary Information (PDF 1, 504 KB)
