# Supplementary material for: Fibrous MXene Aerogels with Tunable Pore Structures for High-Efficiency Desalination of Contaminated Seawater
Source: Nanomicro Lett. 2023 Mar 21;15:71. doi: 10.1007/s40820-023-01030-8 (PMC10030714; doi:10.1007/s40820-023-01030-8)
Supplement: Supplementary file 1 — Supplementary Information (PDF 1, 504 KB) [file 40820_2023_1030_MOESM1_ESM.pdf]

Supporting Information for

Fibrous MXene Aerogels with Tunable Pore Structures for High-Efficiency Desalination of Contaminated Seawater

Fan Wu[1], Siyu Qiang[1], Xiao-Dong Zhu[2], Wenling Jiao[1], Lifang Liu[1], Jianyong Yu[1,*], Yi-Tao Liu[1,*], Bin Ding[1]

[1]Innovation Center for Textile Science and Technology, College of Textiles, Donghua University, Shanghai 201620, P. R. China

[2]State Key Laboratory Base of Eco-Chemical Engineering, College of Chemical Engineering, Qingdao University of Science & Technology, Qingdao 266042, P. R. China

*Corresponding authors. E-mail: liu-yt03@dhu.edu.cn (Yi-Tao Liu), yujy@dhu.edu.cn (Jianyong Yu)

S1 Supplementary Methods

S1.1 Simulated Seawater Preparation

26.5 g of sodium chloride (NaCl), 24 g of magnesium chloride ($MgCl_2$), 0.73 g of potassium chloride (KCl), 3.3 g of magnesium sulfate ($MgSO_4$), 0.2 g of sodium bicarbonate ($NaHCO_3$), 1.1 g of calcium chloride ($CaCl_2$), and 0.28 g of sodium bromide (NaBr) were mixed with 1 L of deionized water to obtain simulated seawater with a pH of 7.02.

S1.2 Characterizations

SEM was carried out by a Hitachi S-4800 microscope. TEM was carried out by a JEOL JEM-2100F microscope. AFM was carried out by a Bruker MM8 microscope. Optical photos were recorded by an Olympus BH2-UMA microscope. XRD was carried out by an Advance D8 diffractometer with Cu Kα radiation (λ = 1.5406 Å). XPS was carried out by a ULVAC-PHI Quantera SXM spectrometer. Raman was carried out by a Renishaw spectrometer (λ = 633 nm). UV-vis-NIR was carried out by a Shimadzu UV-3600 spectrophotometer. The compression–recovery and buckling–recovery tests were performed by a DMA (Q850, TA Instruments). The in-situ bending test was performed by an FIB-SEM (Crossbeam340, Carl Zeiss). The underwater oil contact angle was recorded by a contact angle goniometer (SL 200B, Kino). The thermal conductivity was recorded by a Hot Disk instrument (TPS2500S, Hot disk). The infrared images were captured by an infrared camera (TiS75, Fluke). The crystallization temperature was measured by a DSC (200 F3, Netzsch). The concentrations of salt ions were measured by ICP-AES (Leeman Prodigy).

S2 Supplementary Figures

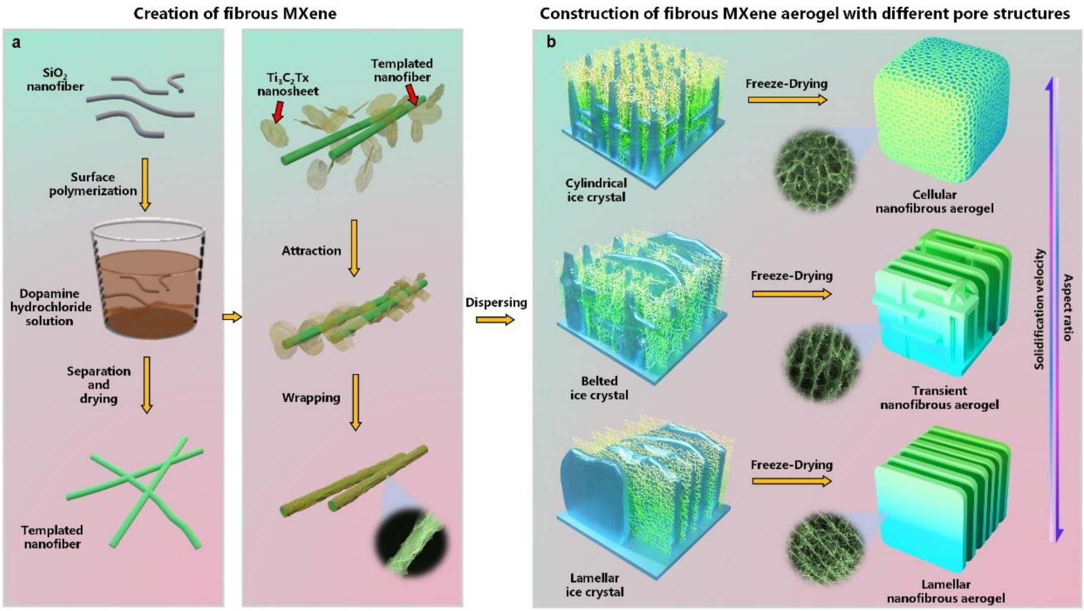

Scheme S1 a Creation of 1D FMs. **b** Construction of FMAs with tunable pore structures

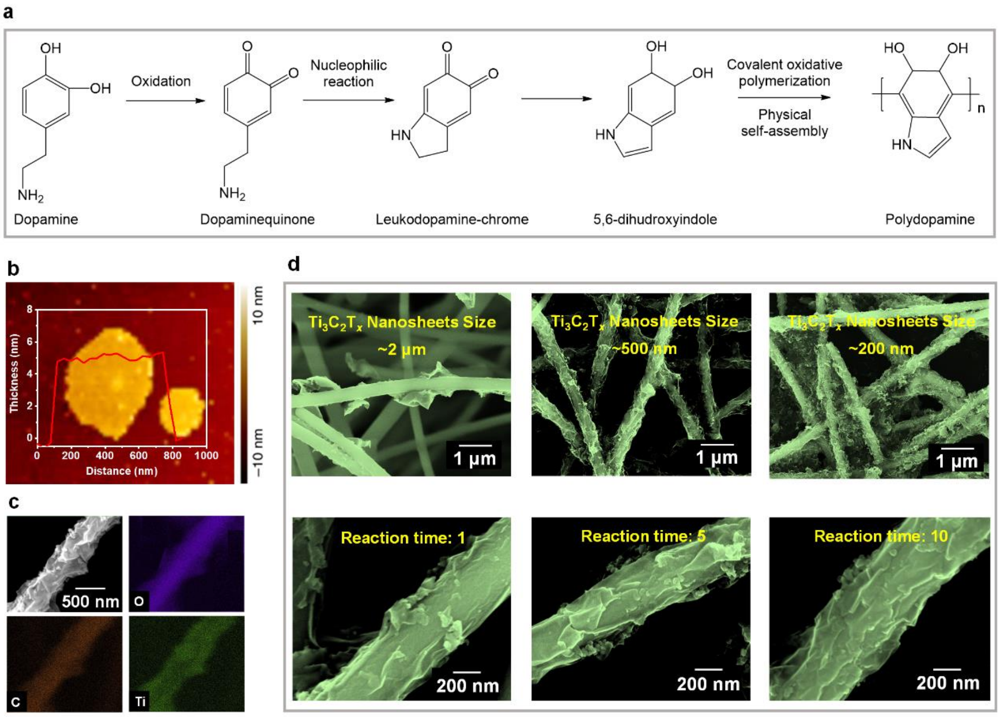

Fig. S1 a Polymerization reaction of dopamine. **b** AFM images of $Ti_3C_2T_x$ nanosheets. **c** EDS maps of an individual FM. **d** SEM images of FMs with different sizes of $Ti_3C_2T_x$ nanosheets and reaction times

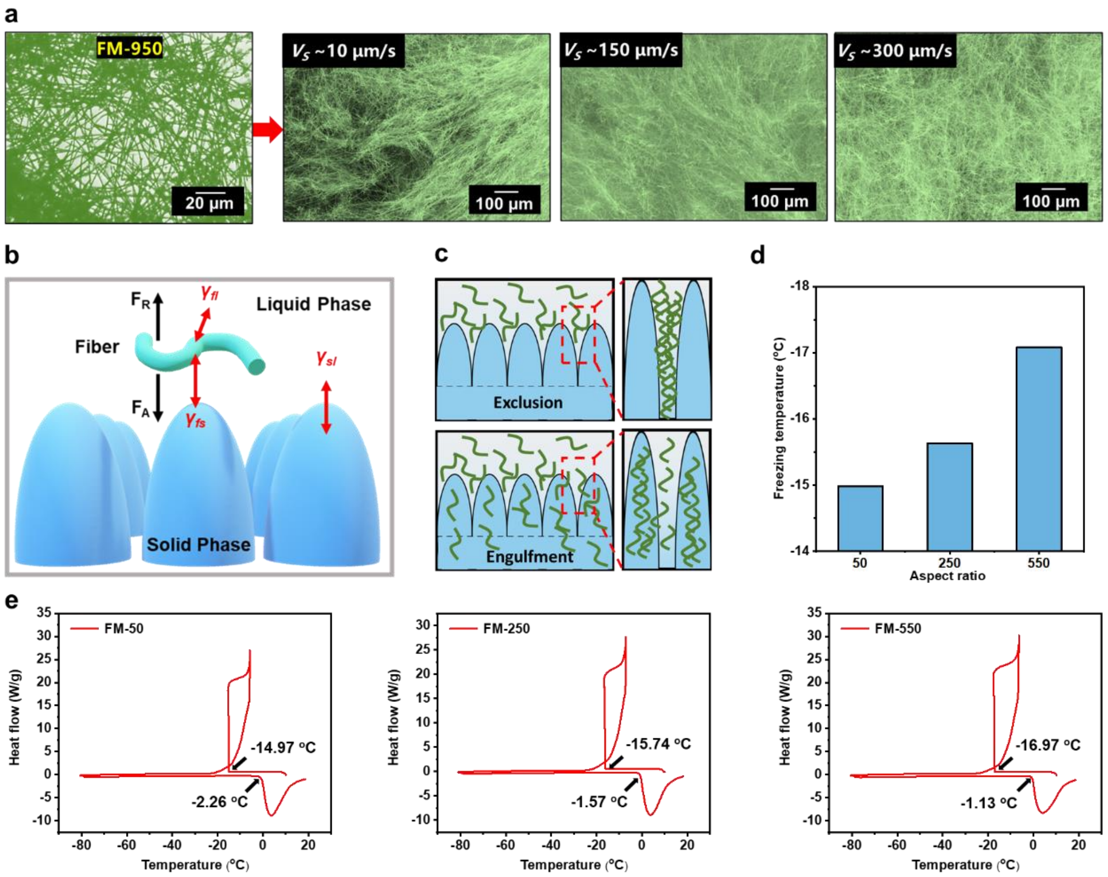

Fig. S2 a Pore structures of FMAs constructed from FM-950 by regulation of solidification velocity. **b** Schematic illustration of forces acting on an individual FM in the vicinity of a freezing front. **c** Schematic illustration of exclusion or engulfment state of FMs. **d** Comparison of freezing temperatures of FMs with different aspect ratios. **e** DSC thermograms of FM-50, FM-250, and FM-550 dispersions during the cooling process

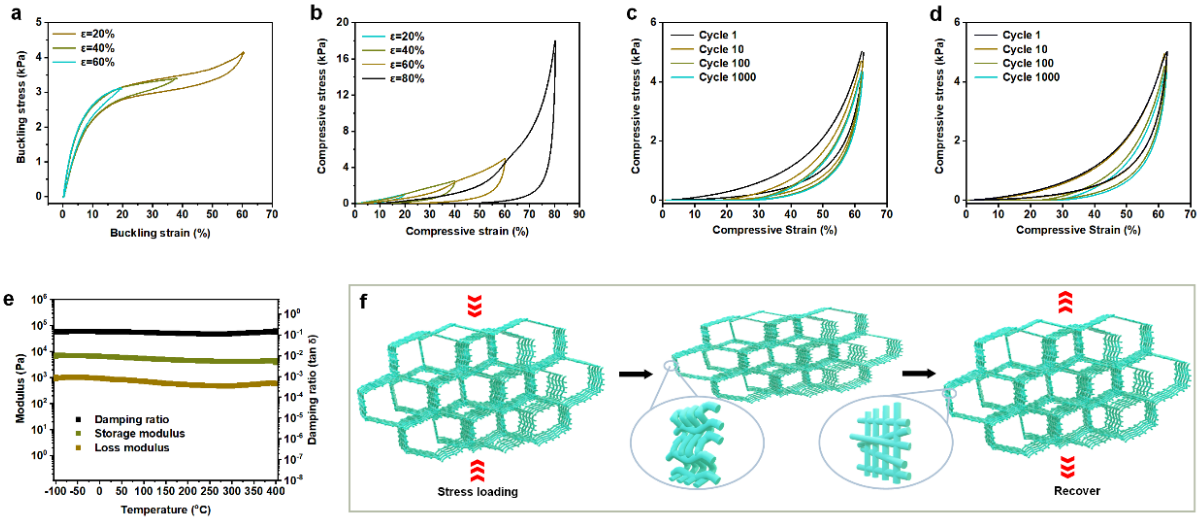

Fig. S3 a Buckling stress–strain curves of *l*-FMAs with increasing strain (0 to 60%). **b** Compressive stress–strain curves of *l*-FMAs with increasing strain (0 to 80%). **c** 1000-cycle fatigue test with 60% compressive strain of *l*-FMAs. **d** 1000-cycle fatigue test with 60% compressive strain of *c*-FMAs. **e** Temperature dependence of storage modulus, loss modulus, and damping ratio for *c*-FMAs. **f** Schematic models showing the compression–recovery behavior of cellular pore structure

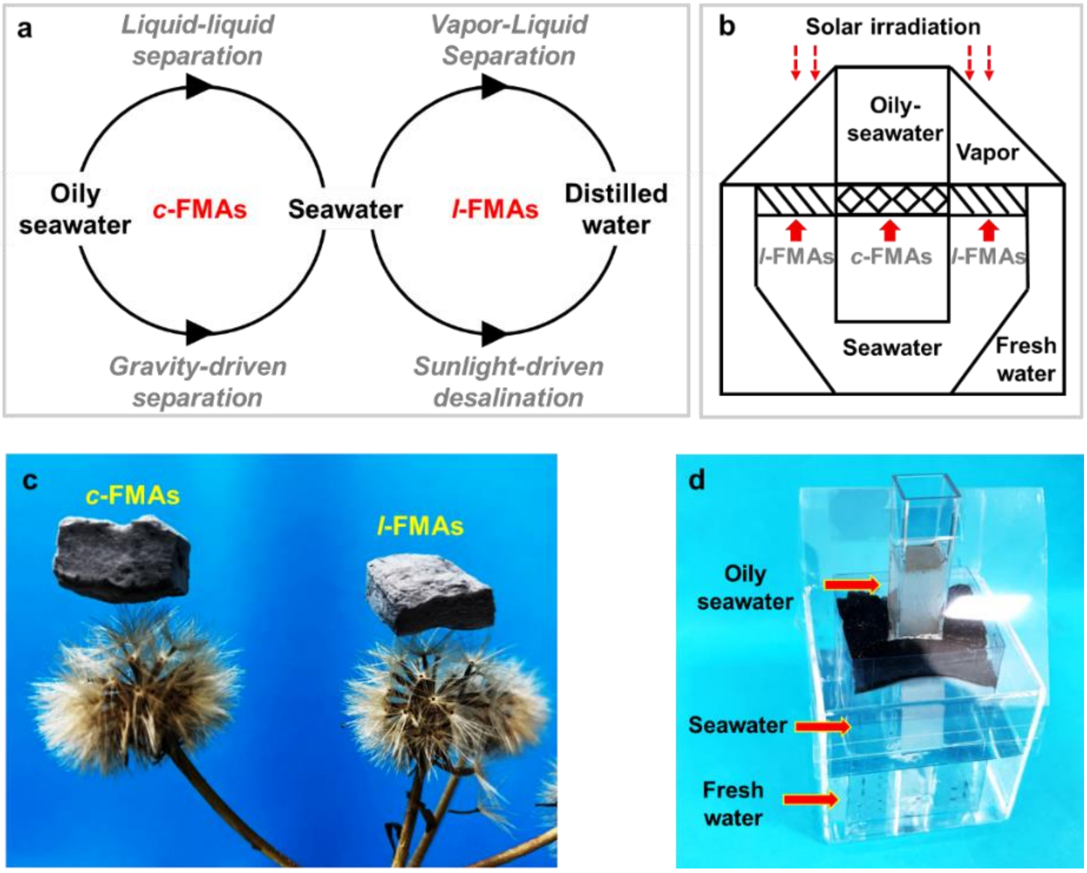

Fig. S4 a and **b** Concept diagram of dual-cycle desalination system. **c** Optical photo of *c*-FMAs and *l*-FMAs. **d** Demo of modularized solar evaporator

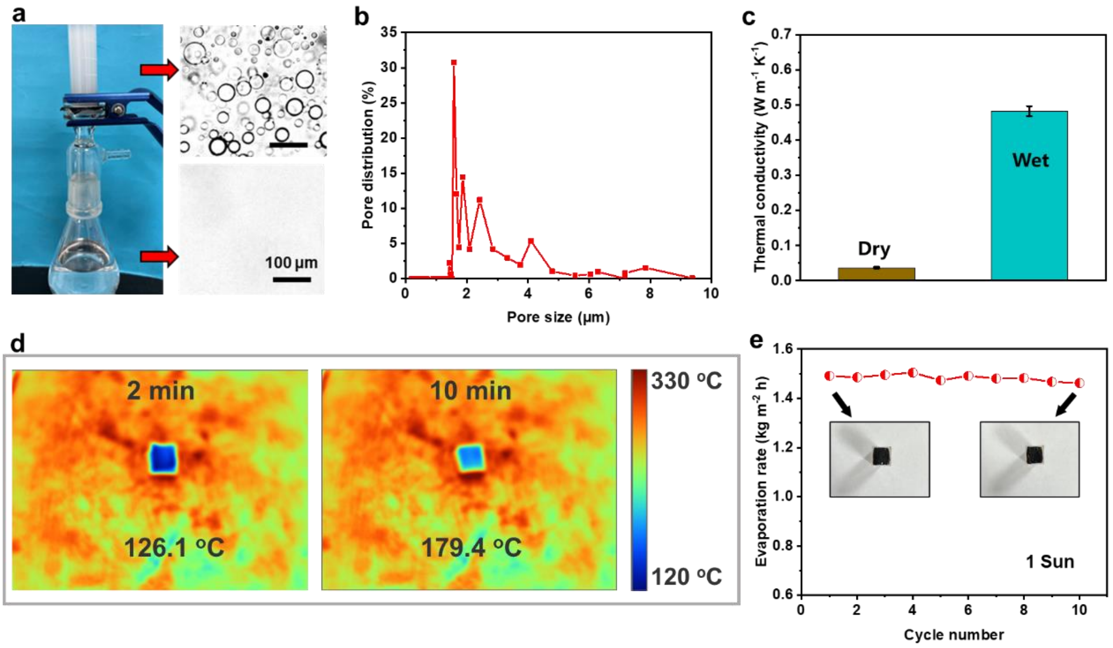

Fig. S5 a Separation apparatus by gravity-driven separation of oil-in-water emulsions using *c*-FMAs, and the corresponding microscopic images of emulsions before and after separation. **b** Pore size distribution of *c*-FMAs. **c** Thermal conductivity of *l*-FMAs in the dry and wet states. **d** Infrared images of *l*-FMAs placed on a hot desk for 10 min. **e** Stability of *l*-FMAs under one sun over 10 cycles (1 h for each cycle). The inset in **e** shows the surface states of *l*-FMAs in the 1st and 10th cycles

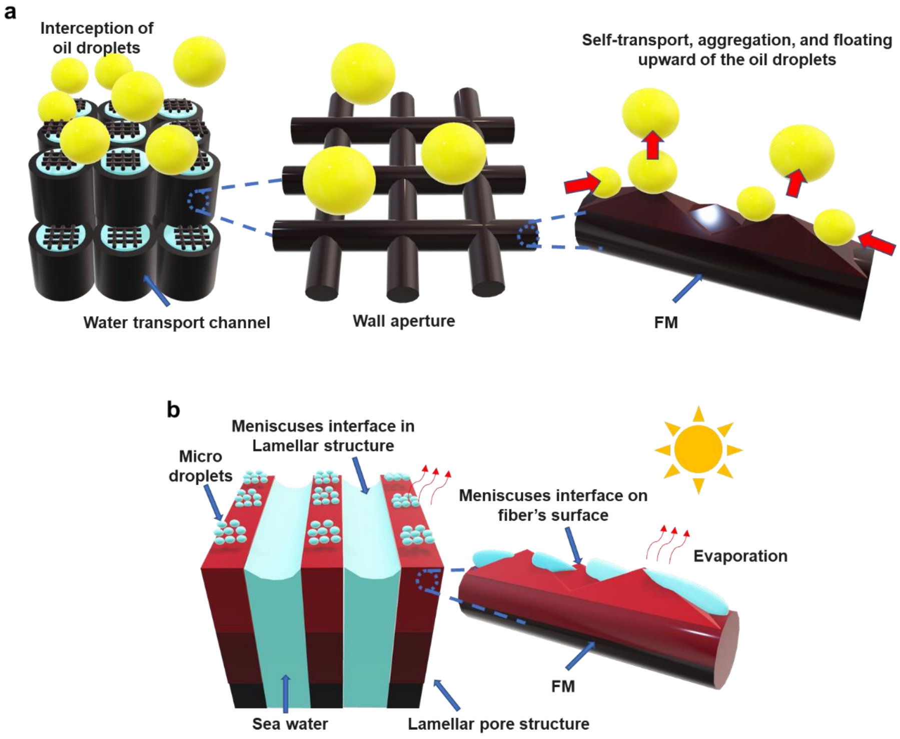

Fig. S6 a Multi-sieving effect in *c*-FMAs. **b** Multi-meniscuses evaporation interfaces in *l*-FMAs

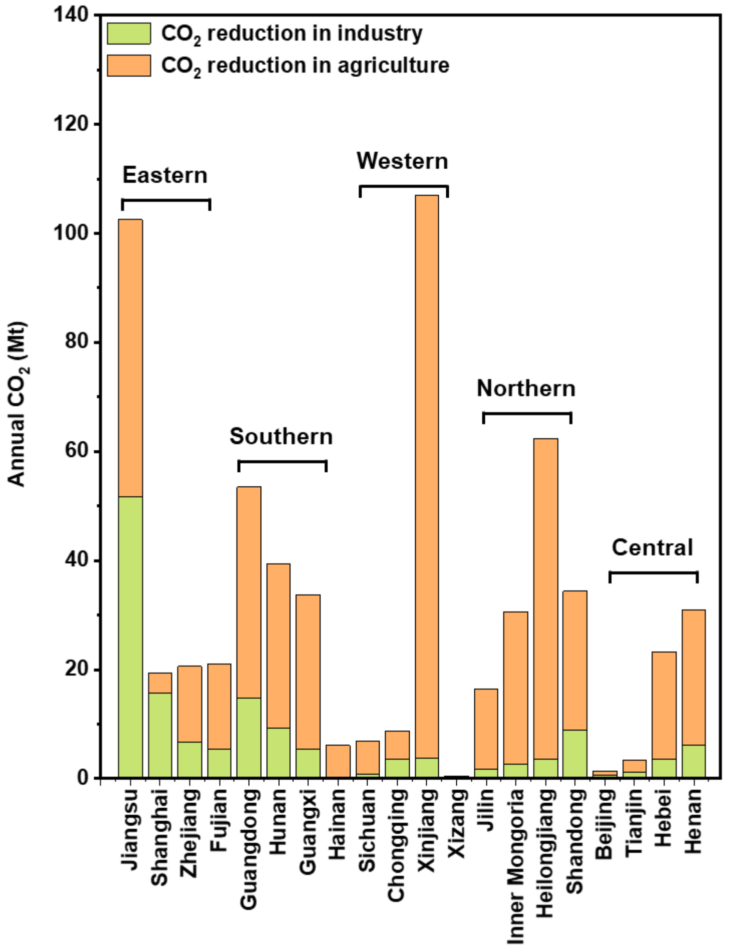

Fig. S7 Annually averaged CO_2 reduction in industry and agriculture over different regions in China as simulated based on our modularized solar evaporator

S3 Supplementary Tables

Table 1

Average aspect ratio	Rotational speed (Shearing, r/min)	Shearing time (min)	Number of cycles	Rotational speed (Centrifugation, r/min)	Centrifugation time (min)
50	10000	1	20	6000	8
250	7000	1	10	4500	8
550	7000	1	6	4500	8
950	3000	1	2	/	/

Table 2

Province	Productivity of thermal power generation (kWh)	Productivity of hydroelectric power generation (kWh)	Productivity of wind power generation (kWh)	Productivity of nuclear power generation (kWh)	Productivity of solar power generation (kWh)	Industrial water consumption (Billion m³)	Agricultural water consumption (Billion m³)
Beijing	444.6	12.8	0..1	0	1.5	2.9	2.8
Tianjin	497.7	0	10	0	6.01	4.8	9.3
Shanghai	454.5	0	8.4	0	0.31	65	15.3
Chongqing	344.1	112.4	8.2	0	2.14	19.3	28.7
Hebei	2222.5	5.9	354.1	0	105.7	17.7	97.1
Henan	1231.7	44.4	95.4	0	25.49	28	115
Yunnan	343.8	2218.6	173.9	0	27.39	15.7	112.1
Liaoning	1350.1	33.9	171.4	358.5	25.7	16.5	77.2
Heilongjiang	850.3	25.2	118.8	0	33.2	17.8	289.2
Hunan	904.4	460.7	125.2	0	15.7	62.1	199.9
Anhui	2690.3	55.7	85.6	0	80.3	82.1	144.1
Shandong	3464	6.3	204.1	134.6	49.59	40.3	115.8
Xinjiang	3014.2	217.5	427.1	0	127.7	18.7	527.9
Jiangsu	4004	26.3	288.8	400.2	68.1	250.2	246.2
Zhejiang	2770.8	146.4	36.5	664.6	47.5	35.8	73.3
Jiangxi	1117.5	60	77	0	38	48.7	167.3
Hubei	978.7	966.7	66.6	0	30.22	85.6	177.7
Guangxi	597.8	251.1	61.8	93.1	5.98	36.5	189.6
Gansu	678.3	213.8	187.5	0	75.58	6.5	82.6
Shanxi	2378.2	25.5	254.5	0	89.46	12.3	40.8
Inner Mongoria	3639.3	36.2	617.2	0	106.19	13.4	137.5
Shaanxi	2041.4	110.7	126.4	0	74.6	10.9	54.6
Jilin	483.5	62	80.2	0	19.98	9.2	79.9
Fujian	1711	181.1	134.9	777.2	4	35.4	99.8
Guizhou	748	335.5	45.6	0	27.03	20	62.1
Guangdong	2220.2	64.6	46.8	576.8	21.21	78.2	204.2
Qinghai	114.4	402.5	82.2	0	135.3	2.5	17.5
Xizang	3.4	68	0	0	7.9	1.1	27.3
Sichuan	663	106.2	106.2	0	28.8	21.8	158.6
Ningxia	1596.9	20.5	249.7	0	140.3	4.2	56.9
Hainan	227	7.9	4.3	89.4	5.5	1.5	34

S4 Supplementary Note (Note S1)

Potentially, we demonstrated the simulation application of the modularized solar evaporator to provide freshwater through gravity-driven separation and photothermal desalination. Here, we

collected the data on power generation structure and industrial-agricultural water consumption over 31 different provinces in China 2021, and used the reverse osmosis technology as a comparison to calculate the CO_2 reduction potential. The following calculation algorithm was constructed to estimate total CO_2 reduction with the modularized solar evaporator:

$$R_{co_2} = E_{pco_2} P\, C_w \eta_{ec} - E_{modularized\ solar\ evaporator}$$

Where E_{Eco_2} and p were CO_2 emissions per kWh of electricity and weighted power generation over 31 provinces. C_w and η_{ec} were water consumption over 31 provinces and electricity consumption for water purification through reverse osmosis technology.

Supplementary References

[S1] J. Zhao, K. Yu, Y. Hu, S. Li, X.Tan et al., Discharge behavior of Mg–4 wt% Ga–2 wt% Hg alloy as anode for seawater activated battery. Electrochim. Acta **56**, 8224-8231 (2021). https://doi.org/10.1016/j.electacta.2011.06.065

[S2] S. Ahuja, *Comprehensive Water Quality and Purification*. (Elsevier Amsterdam, 2014). https://doi.org/10.1016/b978-0-12-382182-9.00001-3
